# Prevalence and prognostic impact of HPV, EBV, and HIV in head and neck squamous cell carcinoma in the Brazilian Amazon cohort

**DOI:** 10.1007/s12094-026-04243-6

**Published:** 2026-02-05

**Authors:** Alberto Mitsuyuki de Brito Kato, Francisco Cezar Aquino de Moraes, Valdenira de Jesus Oliveira Kato, Daniel Oliveira Kato, Helder Antônio Rebelo Pontes, Susanne Suely Santos da Fonseca, Eliel Barbosa Texeira, Amanda de Nazaré Cohen-Paes, Diego Di Felipe Avila Alcantara, Rommel Mario Rodriguez Burbano

**Affiliations:** 1 Biological Science Institute, Federal University of Pará, Belem, 66075110 Brazil; 2 Hospital Ophir Loyola, Belém, 66063-240 Brazil; 3 University of Sao Paulo, Sao Paulo, 05508-220 Brazil; 4 University Center of Pará, Belém, 6613-903 Brazil; 5 Oncology Research Center, João of Barros Barreto University Hospital, Federal University of Pará, Belém, PA 66073-005 Brazil

**Keywords:** Squamous cell carcinoma of the head and neck, HPV, EBV, HIV, TP53, EGFR

## Abstract

**Background:**

Squamous cell carcinoma of the head and neck (SCCHN) is the fifth most common cancer in the world. We investigate the general frequency of SCCHN in the Brazilian Amazon to identify the prevalence of HPV, EBV, and HIV among the local population by means of PCR and to examine the evolution and prognosis of patients.

**Methods:**

This study included 190 individuals with SCCHN and was conducted in the outpatient and inpatient units of the Surgical Clinic of the Department of Head and Neck Surgery of a Hospital in the Brazilian Amazon. We performed HPV detection through PCR and sequencing, EBV detection through RNA in situ hybridization (ISH), and HIV detection through RNA amplification. Statistical analysis included survival estimates through the Kaplan‒Meier curve.

**Results:**

Most participants were male (77.9%, 95% CI 72.0–83.8), while 22.1% (95% CI 16.2–28.0) were female. The mean age was 62.2 years (± 12.6; 95% CI 60.4–63.9), with a median age of 64.0 years (range: 27.0–89.0; 95% CI 60.4–63.9). EBV was not significantly associated with SCCHN and may have only been a contaminant at the evaluated sites. Individuals with mutations in the *TP53* and *EGFR* genes developed more aggressive cancer phenotypes, leading to a 2.6-fold increase in the risk of death. SCCHN was present in the sample, affecting 3.5 times more men than women, with stage IV being the most frequent.

**Conclusions:**

TP53 and EGFR gene mutations were associated with more aggressive cancer phenotypes, leading to a 2.6-fold increase in the risk of death.

## Introduction

Head and neck squamous cell carcinoma (HNSCC) is the sixth most common malignant tumor and the eighth leading cause of cancer-related deaths worldwide, primarily affecting men in their sixth decade of life [1–3]. This neoplasm predominantly involves the squamous cells of the mucosa in the head and neck regions, including mouth, pharynx, larynx, and esophagus [1, 4]. Several risk factors are associated with this neoplasm, such as alcohol and tobacco consumption and viral infections, including oncogenic human papillomavirus (HPV) [5], Epstein–Barr virus (EBV) [6], and human immunodeficiency virus (HIV) [7]. HNSCC is the third most common head and neck malignancy in patients living with HIV, surpassed only by Kaposi's sarcoma (KS) and non-Hodgkin lymphoma [8–10].

These viral infections play roles in the tumorigenesis of SCCHN because they can inactivate tumor suppressor proteins and stimulate cell proliferation, leading to the formation of precancerous lesions that can progress to cancer. This process can occur in patients with SCCHN who are diagnosed with HPV [11]. HPV contributes to oncogenesis in HNSCC through the tumor suppressor gene TP53 [12–14] and epidermal growth factor receptor (EGFR) [15, 16]. For example, the TP53 gene, which is located on chromosome 17p13 and encodes the p53 protein, plays a crucial role as a tumor suppressor in several types of cancer. In SCCHN, it is frequently inactivated, mainly by missense mutations combined with allelic loss. Somatic mutations in TP53 are found in 60–80% of SCCHN/HPV-negative cases [16].

Growing evidence has confirmed the risk relationship between oncogenic HPV infection and SCCHN in the general population. Some studies have shown that HPV-positive SCCHN patients are often associated with advanced tumors and basaloid morphology; however, this characteristic appears to substantially improve survival [7]. Thus, SCCHN exhibits a “new” epidemiological profile when HPV is introduced into the etiopathogenesis of this cancer. The aim of this study was to determine the prevalence of HPV, EBV, and HIV among patients with head and neck carcinoma in Brazil to devise new treatment strategies, improve quality of life, avoid excessive treatment (overtreatment) and reduce public health costs.

## Materials and methods

### Patients and sample collection

This study was conducted in the outpatient and inpatient units of the Surgical Clinic of the Department of Head and Neck Surgery of a Hospital in the Brazilian Amazon, located in Belém do Pará, Brazil; Is a public teaching hospital within the Brazilian Unified Health System (SUS) and a reference center for oncology in the capital of the state of Pará. Samples were collected from 190 individuals with SCCHN, with histological confirmation performed by an experienced pathologist from the referral hospital.

The patients, of both sexes, were treated at the outpatient unit and at the surgical clinic from 2012 To 2016. The inclusion criteria were as follows: 1. Patients with stages I, II, III and IV SCCHN in the oral cavity, pharynx (oropharynx and hypopharynx) and larynx; 2. Patients undergoing surgical treatment and/or chemotherapy and/or radiotherapy; 3. Patients above 18 years of age who underwent outpatient follow-up; and 4. Patients who signed an informed consent form.

The exclusion criteria were as follows: 1. Patients who had not undergone staging; 2. Patients who abandoned treatment for any reason; 3. Patients with synchronous tumors; 4. Patients with incomplete medical records; 5. Patients lost to clinical follow-up; 6. Patients from a state outside the Amazon region; and 7. Patients who did not sign an informed consent form.

For staging, the tumor, lymph node, and metastasis (TNM) staging system was used. For patients with histopathologically confirmed SCCHN (International Classification of Diseases 10th Revision), the following predictive variables were evaluated: HPV, HPV type, EBV, HIV, age, sex, location, TNM stage, degree of cell differentiation, alcohol consumption, smoking, overall survival, family history of SSCHN, and *PD53* and *EGFR*.

Histological material was collected. Two tumor samples (at least 0.2 cm^3^) were obtained under local or general anesthesia depending on the stage of the lesion. Subsequently, one sample was fixed in formaldehyde and then paraffinized and analyzed to confirm SCCHN by pathologists at the hospital. The second sample was sent to the Genetics and Molecular Biology Laboratory of the hospital for analysis after confirmation of the pathology. All patient procedures were conducted in accordance with the guidelines and regulations of the Helsinki Declaration. This study was approved by the Research Ethics Committee of Ophir Loyola Hospital (no. 3.244.874).

### HPV analysis by direct sequencing

DNA extraction efficiency was evaluated by PCR amplification of the β-globin gene. To detect HPV, viral gene-specific reactions were performed with the GP5 + and GP6 + primers, which amplify a 150-bp fragment of the L1 viral gene, corresponding to a conserved region of the virus genome. Viral identification was performed by direct sequencing of the PCR product using a 3500 Genetic Analyzer (Thermo Fisher Scientific).

The DNA sequences obtained were subjected to similarity analysis using the BLAST (Basic Local Alignment Search Tool) platform. A sequence homology threshold of 97% or higher was established as the criterion for identifying a specific HPV subtype. This means that, for a sample to be classified as a given HPV subtype, its L1 sequence must exhibit at least 97% identity with a known reference sequence of that subtype in the database. Sequences with homology below 97% were considered “non-typeable” or “undefined” for subtype classification purposes, although the presence of viral DNA was still confirmed.

### Detection of EBV

The presence of EBV in SCCHN samples was detected using a primer with a biotinylated probe (5'-AGACACCGTCCTCACCACCCGGGACTTGTA-3'), which is complementary to EBV-encoded small RNA-1 (Eber1). RNA in situ hybridization (ISH) was used to detect EBV, and a mouse anti-biotin antibody (DakoCytomation®, Carpinteria, CA, USA) and an anti-biotinylated immunoglobulin antibody were used for signal amplification (DakoCytomation®, Carpinteria, CA, USA). Streptavidin–biotin peroxidase complex (DakoCytomation®, Carpinteria, CA, USA) and the chromogen diaminobenzidine (DakoCytomation®, Carpinteria, CA, USA) were used for visualization. The slides were counterstained with Harris hematoxylin, and the cells were examined under a light microscope at 40 × magnification. Ten representative microscopic fields containing at least five cells were evaluated. Epithelial cells were considered positive for EBV when 5% or more cells were stained brown/red. Tumor and non-neoplastic samples from the same patient were analyzed.

### HIV detection

Viral RNA was extracted using a QIAGEN (Venlo, the Netherlands) QIAamp Viral RNA Mini Kit and transferred to a COBAS TaqMan HIV-1 Test, v2.0 (Roche, Pleasanton, CA) for amplification and detection.

### Mutation analysis of the TP53 gene by nucleotide sequencing

The *TP53* gene has 11 exons. In the human p53 protein, exon 1 is non-coding and thus was not analyzed in this study. Exons 2–11 of the *TP53* gene were amplified separately by PCR using the specific *primer* sets described by Donehower et al. [17]. Direct sequencing of the amplified products was performed using the ABI Prism 3130 (Thermo Fisher Scientific, USA) automatic sequencer. The resulting sequences were directly edited in the Sequencing Analysis Software on a computer connected to a 3730XL DNA Analyzer (Thermo Fisher Scientific, USA).

#### *Fluorescence *in situ* hybridization (FISH)*

Only tumor samples with*TP53* gene mutations were analyzed by FISH. To determine the number of copies of chromosome 17 and of the *TP53* gene, the cells were hybridized using a directly labeled probe (Abbott/Vysis, USA) specific for chromosome 17 α-satellite (SpectrumGreen; p11.1-q11.1) and the *TP53* gene region (SpectrumOrange; 17p13.1). Nuclei were counterstained with DAPI/antifade. For each tumor, 200 interphase nuclei were counted and analyzed using the criteria described by Hopman et al. [18].

### Statistical analysis

In the first stage, descriptive statistical analysis generated absolute and relative distributions of the epidemiological, clinical, and molecular variables selected for the study. Univariate analysis identified variables to highlight among all cancer patients.

In the second stage, descriptive bivariate results were evaluated using chi-square statistical test to determine any influences on the variables identified. Survival estimates were established using the Kaplan‒Meier estimator, which yielded an empirical estimate for survival data.

Log-rank test was used to compare the survival curves, and the Akaike information criterion (AIC) was used to select covariates to build a model with the highest goodness of fit.

## Results

### Study population

**Fig. 1:**
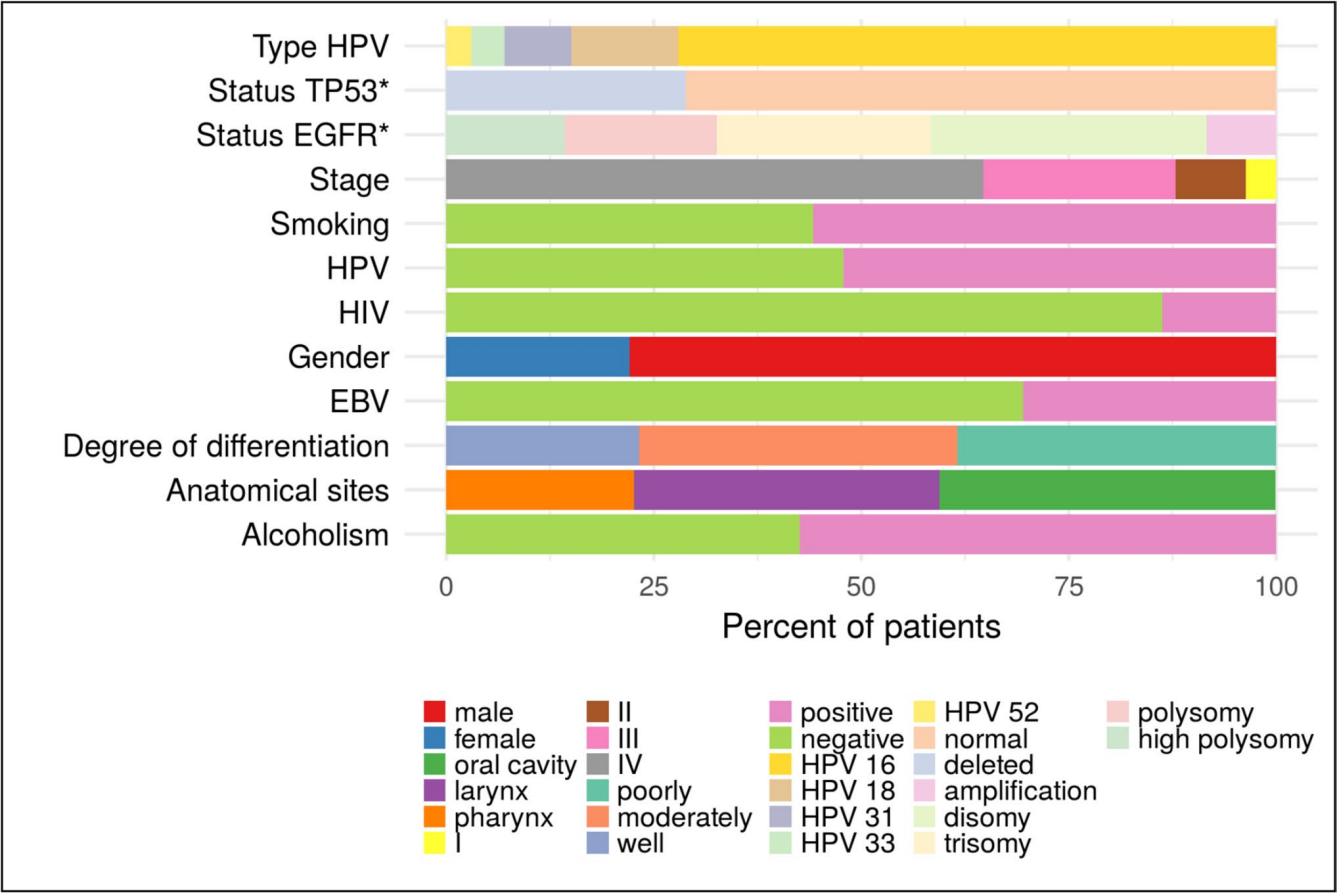
Sample Distribution by Category. The bar plot was constructed based on the percentage of patients, where each horizontal line represents the investigated categories, and the colors represent the subcategories

This study analyzed demographic, clinical, and virological characteristics in 190 included patients. Most participants were male (77.9%, 95% CI 72.0–83.8), while 22.1% (95% CI: 16.2–28.0) were female. The mean age was 62.2 years (± 12.6; 95% CI 60.4–63.9), with a median age of 64.0 years (range: 27.0–89.0; 95% CI 60.4–63.9). Regarding disease stage, 5.3% (95% CI 2.1–8.4) were diagnosed at stage I, 27.4% (95% CI 21.0–33.7) at stage II, 34.2% (95% CI 37.5–41.0) at stage III, and 34.2% (95% CI 27.5–41.0) at stage IV. Concerning tumor histological differentiation, 23.2% (95% CI 17.2–29.2) were well differentiated, 38.4% (95% CI 31.5–45.3) moderately differentiated, and 38.4% (95% CI 31.5–45.3) poorly differentiated. EBV status was positive in 30.5% (95% CI 24.0–37.1) and negative in 69.5% (95% CI 62.9–76.0). Regarding HPV infection status, the most prevalent subtype was HPV16 (37.9%, 95% CI 31.0–44.8), followed by HPV18 (6.8%, 95% CI: 3.3–10.4), HPV31 (4.2%, 95% CI 1.4–7.1), HPV33 (2.1%, 95% CI 0.1–4.1), and HPV52 (1.6%, 95% CI 0.2–3.4), while 47.4% (95% CI 40.3–54.5) tested negative for the viral infection. HIV status was positive in 13.7% (95% CI 8.8–18.6) and negative in 86.3% (95% CI 81.4–91.2). A history of alcoholism was identified in 57.4% (95% CI 50.3–64.4), while 42.6% (95% CI 35.6–49.7) reported no history. Among the 190 patients, 55.8% (95% CI 48.7–62.9) were smokers, while 44.2% (95% CI 37.1–51.3) were non-smokers (Table 1 and Fig. 1).

**Table 1:** Demographic, Clinical, and Virological Characteristics of the Study Population

Variables	Frequency (%)	CI 95%
Gender		
Female	42 (22.1)	16.2–28.0
Male	148 (77.9)	72.0–83.8
Age		
Average	62.2 (± 12.6)	60.4–63.9
Median	4.0 (27.0–89.0)	60.4–63.9
Stage		
I	10 (5.3)	2.1–8.4
II	52 (27.4)	21.0–33.7
III	65 (34.2)	37.5–41.0
IV	63 (34.2)	27.5–41.0
Degree of differentiation		
Well	44 (23.2)	17.2–29.2
Moderately	73 (38.4)	31.5–45.3
Poorly	73 (38.4)	31.5–45.3
EBV		
Positive	58 (30.5)	24.0–37.1
Negative	132 (69.5)	62.9–76.0
HPV Status		
HPV16	72 (37.9)	31.0–44.8
HPV18	13 (6.8)	3.3–10.4
HPV31	8 (4.2)	1.4–7.1
HPV33	4 (2.1)	0.1–4.1
HPV52	3(1.6)	0.2–3.4
Negative	90 (47.4)	40.3–54.5
HIV		
Positive	26 (13.7)	8.8–18.6
Negative	164 (86.3)	81.4–91.2
Alcoholism		
Yes	109 (57.4)	50.3–64.4
No	81 (42.6)	35.6–49.7
Smoking		
Yes	106 (55.8)	48.7–62.9
No	84 (44.2)	37.1–51.3

*EBV* Epstein–Barr virus; *HPV* Human papillomavirus; *HIV* Human immunodeficiency virus

### Association of EBV, HIV, and EBV in the tumor differentiation stage

Table 2 provides the results of the chi-square analysis of the variables and HPV, EBV, and HIV. There was an association between HPV and tumor stage and differentiation (*p* = 0.01 and *p* = 0.00). Among HPV-negative patients, stage IV was the most predominant, indicating that HPV influences the staging of the disease (*p* = 0.01). In addition, poor differentiation was the most common grade among patients with HPV-positive cancer, suggesting an association between HPV and tumor differentiation grade (*p* = 0.001). Regarding EBV, the variables sex, anatomical site, and degree of tumor differentiation were significantly associated (*p* = 0.01). In addition, there was an association between HIV and anatomical site, tumor stage, and degree of differentiation (*p* = 0.014, *p* = 0.02, and *p* = 0.01) as seen in Table 2.

**Table 2:** Association between viruses (HPV, EBV, and HIV) and clinical variables

		HPV					EBV					HIV				
Variables		Positive	%	Negative	%	(*p*-value)*	Positive	%	Negative	%	(*p*-value)*	Positive	%	Negative	%	(*p*-value)*
Gender	Female	18	9.47	24	12.63	0.17	6	3.16	36	18.95	**0.01**	4	2.11	38	20.00	0.46
	Male	82	43.16	66	34.74		52	27.37	96	50.53		22	11.58	126	66.32	
Anatomical sites	Oral cavity	40	21.05	37	19.47	0.46	18	9.47	59	31.05	**0.01**	17	8.95	60	31.58	**0.014**
	Larynx	34	17.89	36	18.95		19	10.00	51	26.84		4	2.11	66	34.74	
	Pharynx	26	13.68	17	8.95		21	11.05	22	11.58		5	2.63	38	20.00	
Stage	I	5	2.63	2	1.05	**0.01**	4	2.11	3	1.58	**0.29**	0	0.00	7	3.68	**0.02**
	II	10	5.26	6	3.16		4	2.11	12	6.32		0	0.00	16	8.42	
	III	31	16.32	13	6.84		16	8.42	28	14.74		2	1.05	42	22.11	
	IV	54	28.42	69	36.32		34	17.89	89	46.84		24	12.63	99	52.11	
Degree of differentiation	Poorly	49	25.79	24	12.63	**0.001**	32	16.84	41	21.58	**0.01**	18	9.47	55	28.95	**0.01**
	Moderately	28	14.74	45	23.68		13	6.84	60	31.58		8	4.21	65	34.21	
	Well	23	12.11	21	11.05		13	6.84	31	16.32		0	0.00	44	23.16	

*Chi-Square Test (χ.^2^): Chi-square test value to assess the association between the virus and clinical variables, considering statistically significant at *p* ≤ 0.05

### Mutational status of TP53 and EGRF

The highest mean levels of *EGFR* gene quantification (1.67 ± 0.43) were significantly associated (*p* < 0.001) with a greater number of deaths. In addition, there was a significant difference in alterations in the *TP53* and *EGFR* genes (*p* < 0.001). *TP53* gene deletion and *EGFR* gene changes were more frequent in the deceased group than in the survival group (38.7% and 10.61%).

All genetic variables analyzed were associated with the risk of death in patients with head and neck cancer. Quantitative changes in *EGFR* expression and changes in the *TP53* and *EGFR* genes were associated with a significant risk of death (OR = 134.4; 95% CI 27.4–657.0; OR = 5.39; 95% CI  2.27–12.78; OR = 6.68; 95% CI  3.42–13.07, respectively).

### Seropositivity for HPV and HIV, genetic alterations in the TP53 gene, and alterations in the EGFR gene and quantification

Controlled logistic regression analysis was performed for factors that were identified as significant in the first analysis, and the following factors were still significant after controlling for the variables: sero-positivity for HPV and HIV, genetic changes in the TP53 gene, and changes in the EGFR gene and quantification.

Sero-positivity for HPV was a protective factor against death in patients with head and neck cancer (OR = 0.021, 95% CI 0.004–0.10). HIV sero-positivity was a risk factor for death (OR = 279.64, 95% CI  13.22–5912.15).

Alteration in the TP53 gene was a risk factor for mortality, with an OR of 4.36 and a 95% CI of 1.49–12.76. Quantitative changes in the EGFR gene had an OR of 25.68 and a 95% CI of 4.20–156.99. Changes in the EGFR gene, when added together, increased the risk by 2.60, with a 95% CI of 1.11–6.09.

**Fig. 2:**
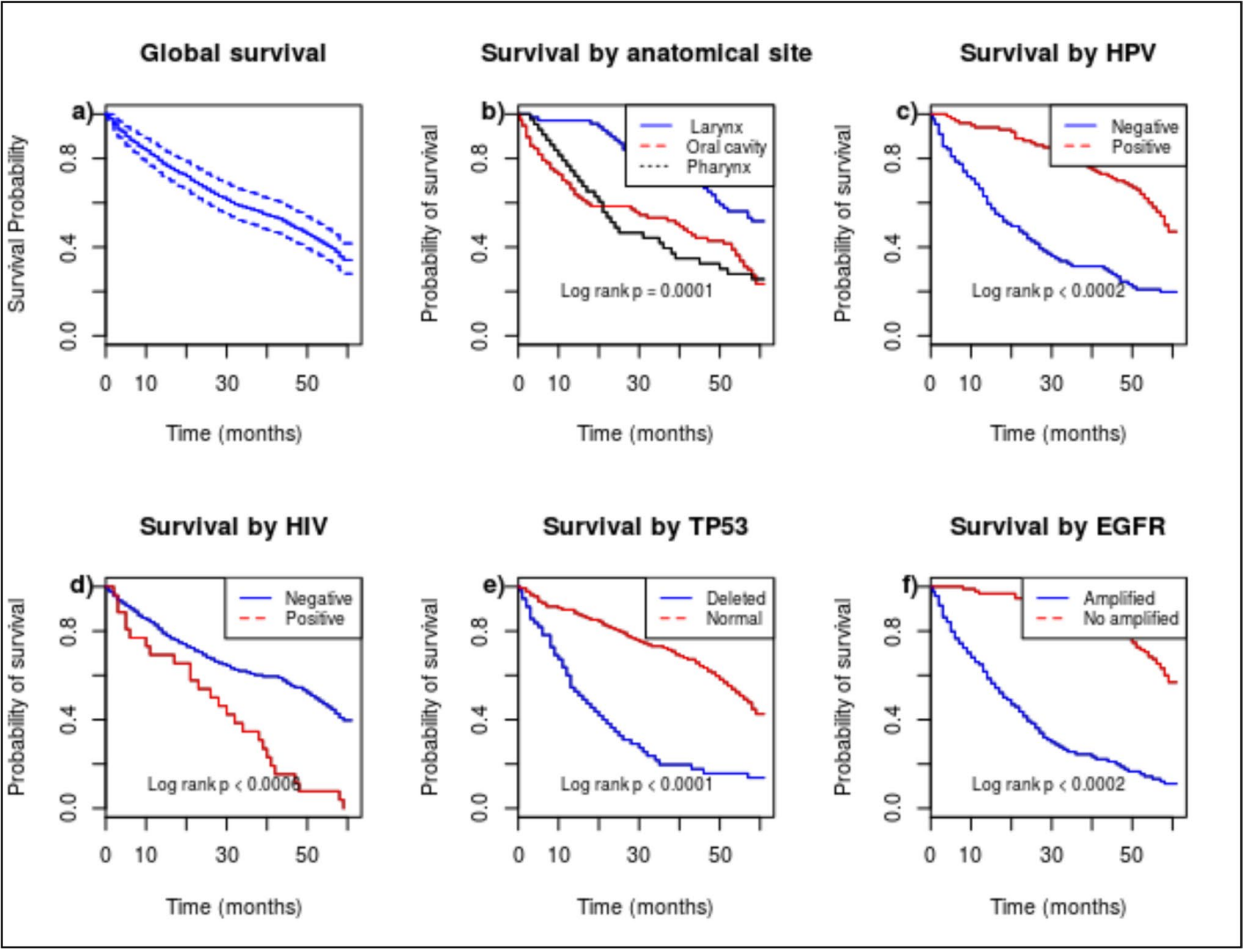
Comparison of Global Survival and Survival by Variable. Kaplan‒Meier survival curve illustrating the probability of survival over time for a group of patients with head and neck cancer. The graph axes display time in months on the x-axis and the probability of survival on the y-axis. Additionally, the log-rank test was performed to compare survival between different groups (2b, c, d, e), with *p* ≤ 0.05 considered statistically significant

Figure 2 provides the univariate logistic regression results for the deceased and survival groups. The patients were followed up for a period of 5 years, and the overall survival rate in this period was 35.3% (Fig. 2a). Tumor location had a significant influence on the survival curve, with laryngeal cancer having the highest survival rate, approximately 55%, and pharyngeal and oral cavity cancers had survival rates of approximately 20%.

In addition, patients with HPV survived longer than did HPV-negative patients (Fig. 2c). Regarding HIV, the survival rate was lower for HIV-positive patients than HIV-negative patients (Fig. 2d). Finally, regarding genetic aspects, patients with *TP53* gene deletion had a 5-year survival rate below 10% (2e).

## Discussion

Most head and neck cancers derived from the mucosal epithelium in oral cavity, pharynx, and larynx and are known collectively as SCCHN [3]. Smoking remains a major risk factor globally, with the rates continuously rising in developing countries [19]. Chronic heavy alcohol consumption is another independent risk factor for head and neck cancers, specifically SCCHN [20], and frequent alcohol consumption also potentiates the carcinogenic effect of tobacco [21].

Additionally, there has been a sudden rise in the incidence of oropharyngeal cancers due to increasing rates of human papillomavirus infection [22]. Of the 120 different human papillomavirus types that have been isolated, oncogenic type 16 (associated with most cases) and oncogenic type 18 account for over 90% of cases of human papillomavirus-related oropharyngeal squamous cell carcinoma [23]. Therefore, SCCHN can be separated into HPV-negative or HPV-positive SCCHN.

In this study, there was an association between HPV and the variables, tumor stage and differentiation. High-risk HPVs, including HPV-16, are small, double-stranded, circular DNA viruses with an ~ 8 kb genome. In SCCHN tumor specimens, the viral genome is typically integrated at a single, albeit variant, genomic site [24]. The genome consists of seven early genes (E1–E7) and two late genes (L1 and L2). The L1 and L2 genes encode viral capsid proteins, and the E1–E5 genes encode proteins that are primarily involved in the replication and transcription of the viral genome. The bicistronic E6 and E7 genes are essential for oncogenic transformation of the host cell. The E6 protein forms a complex with the cellular ubiquitination protein E6-AP and the tumor suppressor p53 to promote the ubiquitination and proteasomal degradation of p53 [25].

Jung et al. (2020) reported that over the past decade, human papillomavirus (HPV) has emerged as a pathogen that causes a distinct group of SCCHN, in particular oropharyngeal squamous cell carcinoma. HPV-related SCCHN affects younger people and has genomic features that are very different from those of HPV-negative SCCHN.

In contrast to HPV-negative SCCHN in which TP53 (encoding p53) is frequently deleted or mutated, in HPV-positive HNSCC, p53 is eliminated by the action of E6 [24, 26]. E6 may possess other transforming activities beyond degradation, but these functions are less well characterized [27–29]. The E7 protein binds strongly to the retinoblastoma-associated protein (RB1) cell cycle regulator, promoting proteasomal degradation of RB1 and the release of E2F family transcription factors [30]. The released E2F proteins drive the cell cycle beyond the G_1_–S checkpoint and into the S phase. E7 also interacts with and affects the levels and/or cell activity of a number of other cell cycle regulatory proteins [27]. The disruption of RB1 function by E7 leads to the feedback upregulation of p16INK4A, and detection of p16INK4A expression is commonly used to classify oropharyngeal tumors as HPV-positive. In addition to E6 and E7, E5 also plays a role in oncogenic transformation by helping to drive cell cycle progression [31].

In addition to TP53, EGFR is widely expressed in head and neck carcinomas [15]. An in vitro study with HPV-positive SCC090, SCC152, and SCC154 cells and HPV-negative SCC072 cells revealed a new perspective on the regulation of HPV E6/E7 proteins in SCCHN/HPV-positive cells, highlighting the role of EGFR, microRNA-9-5p and BRD4 protein in this process. EGFR suppression decreased the expression of HPV E6/E7 proteins, which are known to play important roles in tumorigenesis and the maintenance of SCCHN malignancy, which involves the action of microRNA-9-5p, which negatively regulates the expression of BRD4 protein [15]. These findings may provide valuable insights for the development of new therapeutic strategies for the treatment of HPV-positive SCCHN.

Regarding EBV, the variables, sex, anatomical site, and degree of tumor differentiation were significantly associated (*p* = 0.01). EBV is an infectious agent that is specifically associated with nasopharyngeal carcinoma [32]. For patients with nasopharyngeal carcinoma, plasma DNA testing for EBV, in addition to standard histopathological examinations (including EBV staining of primary tumors), can be used; however, the lack of standardization of such testing severely reduces the reliability of the results [33]. After definitive treatment, circulating EBV DNA has been shown to have promise as a biomarker for surveillance, in addition to its roles in population screening and in determining prognosis [34].

In EBV-positive nasopharyngeal cancer, p53 is downregulated, and miR-BHRF1-1, an miRNA encoded by EBV, contributes to viral lytic replication, favoring the progression of nasopharyngeal carcinoma [21, 35, 36].

These findings reaffirm that viral infections further favor the progression of head and neck cancer. As with the other viruses mentioned, studies have shown that SCCHN occurs at higher rates among people with HIV [7]. In addition, AIDS-cancer registry studies suggest that compared to the general population, HIV-infected individuals have a 1.6 to 6 times greater risk of developing oropharyngeal cancer and a 1.7 to 4 times greater risk of developing head and neck cancer in general [22].

The data suggest an association between HIV and anatomical site, tumor stage and degree of differentiation. Several studies have demonstrated a relationship between HIV and tumor development. Individuals infected with HIV are at increased risk of infection with both low-risk and high-risk HPV types. Chronic immunosuppression provides an environment for persistent HPV infection, in addition to a higher risk of malignant transformation [27]. Very few studies have investigated PLWH and SCCHN. The largest cohort included 286 and 248 patients treated in multiple centers in France and North America, respectively, without an HIV-negative control group as a comparison [7].

For HIV, the high correlation with this neoplasm may be associated with a pattern of mutations that is distinct from that for HIV-negative head and neck cancer. Recent evidence has shown that TP53 mutation frequencies are significantly lower in patients with HIV-related head and neck cancer. Furthermore, mutations in HIV patients tend to be TpC > T nucleotide changes for all mutated genes, especially for the TP53 gene [23].

Regarding genetic factors, the highest mean levels of the *EGFR* gene (1.67 ± 0.43) were significantly associated (*p* < 0.001) with a greater number of deaths. Numerous studies have demonstrated the aberrant expression of signaling proteins and/or the activation of signaling pathways in SCCHN tumors. EGFR is overexpressed in 80–90% of SCCHN tumors and associated with poor overall survival and progression-free survival [37, 38]. Molecularly targeting EGFR with monoclonal antibodies (such as cetuximab) is an FDA-approved strategy for inhibiting EGFR signaling in SCCHN. The overexpression of other receptor tyrosine kinases, including HER2 and MET, also occurs and may contribute to SCCHN resistance to EGFR-targeting agents [39–42].

Some tumors that overexpress EGFR ligands also harbor CCND1 and CDKN2A aberrations, which may render them resistant to anti-EGFR mAb monotherapy. Tumors with high EGFR expression do not necessarily have high levels of EGFR ligands and may not respond to EGFR mAbs. However, these tumors show strong EGFR phosphorylation and thus could respond to small-molecule EGFR tyrosine kinase inhibitors (TKIs) (Huang et al., 2021). Consistent with this hypothesis, patients with SCCHN with p16 negativity and EGFR overexpression obtained clinically meaningful benefit from afatinib, an EGFR-TKI, in a phase III trial [43].

There were significant differences in changes in the *TP53* and *EGFR* genes (*p* < 0.001); *TP53* gene deletion and *EGFR* gene changes were more frequent in the deceased group than in the survival group (38.7% and 10.61%, respectively). Alterations in the *TP53* gene have also been frequently reported in SCCHN. Structural changes (homozygous deletions and intra- and inter-chromosomal fusions) were more commonly associated with loss of function in tumor suppressor genes, most prominently *CDKN2A*, followed by *TP53*, *RB1*, *NOTCH1*, and *FAT1*, than with protein-coding fusion events.

SCCHN is characterized by genetic instability, with frequent loss or gain of chromosomal regions [24]. The availability of a model of ordered histological progression of SCCHN has enabled the assignment of some chromosomal abnormalities to specific stages of progression [44].

Loss of 9p21 occurs during the progression of mucosal epithelial cells of head and neck from normal to hyperplastic. The 9p21 region includes the tumor suppressor genes (TSGs) CDKN2A (which encodes the CDK4 and CDK6 inhibitor p16INK4A) and ARF (which encodes p14, a stabilizer of p53). Progression from hyperplasia to dysplasia is marked by the loss of 3p21 and 17p13, the site of TP53. The transition from dysplasia to carcinoma in situ involves the loss of 11q13, 13q21, and 14q32, and the loss of 6p, 8, 4q27, and 10q23 occurs in the progression to invasive carcinoma. Collectively, these studies of chromosomal abnormalities reveal that multiple genetic abnormalities may be required for full transformation to invasive SCCHN [45]. However, whether the progression of SCCHN is strictly dependent on the temporal sequence of these alterations or, instead, on their collective accumulation must be further investigated.

Traditionally, frequent mutations in SCCHN were thought to be nonactionable as most of the genomic alterations were found in tumor suppressor genes such as TP53. However, a small number of SCCHN cases do harbor distinct oncogenic driver mutations in genes such as HRAS and PIK3CA [26].

An important limitation of this study is that all biological samples and clinical data were obtained from a single public oncology referral hospital in the Brazilian Amazon. While this setting ensures standardized diagnostic procedures, uniform management protocols, and reduced methodological variability, it also constrains the representativeness of the broader SCCHN population in Brazil.

## Conclusion

SCCHN was present in the sample, affecting 3.5 times more men than women, with stage IV being the most frequent. The prevalence of HPV among patients with SCCHN was greater than 56%, approaching the rate observed in economically developed countries. The site with the highest incidence was the oral cavity, a finding that differs from results reported in the international literature, with the oropharynx being the main site of involvement. HPV-positive HPV patients had a better prognosis, unlike HIV-positive patients, who had a worse prognosis. EBV was not significantly associated and may have been just a contaminant at the evaluated sites. TP53 and EGFR gene mutations were associated with more aggressive cancer phenotypes, leading to a 2.6-fold increase in the risk of death.

## Data Availability

The datasets generated and/or analyzed during the current study are available within the manuscript or supplementary information files.
